# De novo recurrent spontaneous coronary artery dissection in nonatherosclerotic elderly woman: A case report

**DOI:** 10.1002/ccr3.3079

**Published:** 2020-07-15

**Authors:** Jun Kozuki, Shigeyasu Tsuda

**Affiliations:** ^1^ Division of Cardiology Kita‐Harima Medical Center Ono Japan

**Keywords:** spontaneous coronary artery dissection

## Abstract

Spontaneous coronary artery dissection (SCAD) is a rare disease which causes acute myocardial infarction (AMI). In this case, we show the recurrence of SCAD and pathological findings of an intimal tear that lead to SCAD of the proximal left anterior descending branch which could contribute to the onset of AMI.

## Graphical abstract

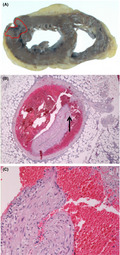

## 1 INTRODUCTION

Spontaneous coronary artery dissection (SCAD) is an important disease causing acute coronary syndrome (ACS) and is defined as a coronary artery dissection that is not traumatic, iatrogenic, and not with atherosclerosis.^1^ Generally, SCAD appears to have a particular predominance in young women or people with less conventional atherosclerotic risk factors.^2^ In pathology, the tunica media is separated from the tunica intima by intramural hemorrhage (IMH), leading to compression of the true lumen. IMH is thought to occur by two mechanisms. The first theory proposes that a disruption of the tunica intima is the initial change, and the second one proposes that a rupture of a single vasa vasorum, which is within the vessel wall and provides the vessel with blood, is the initial change.^2^, ^3^ A single vasa vasorum in atherosclerotic coronary arteries has a different character from that of the normal coronary artery and may be fragile and prone to rupture.^4^ In this report, we detect important pathological findings suggestive of an intimal tear and support the first proposed mechanism of SCAD onset.

## 2 CASE HISTORY

**FIGURE 1:**
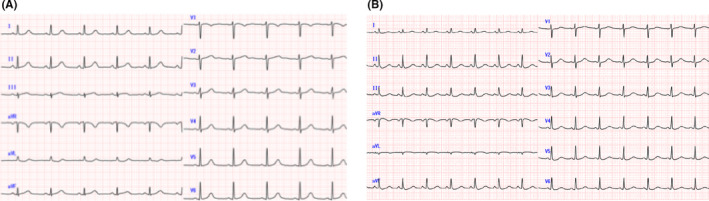
Electrocardiogram in 2015 (A) and 2018 (B)

**FIGURE 2:**
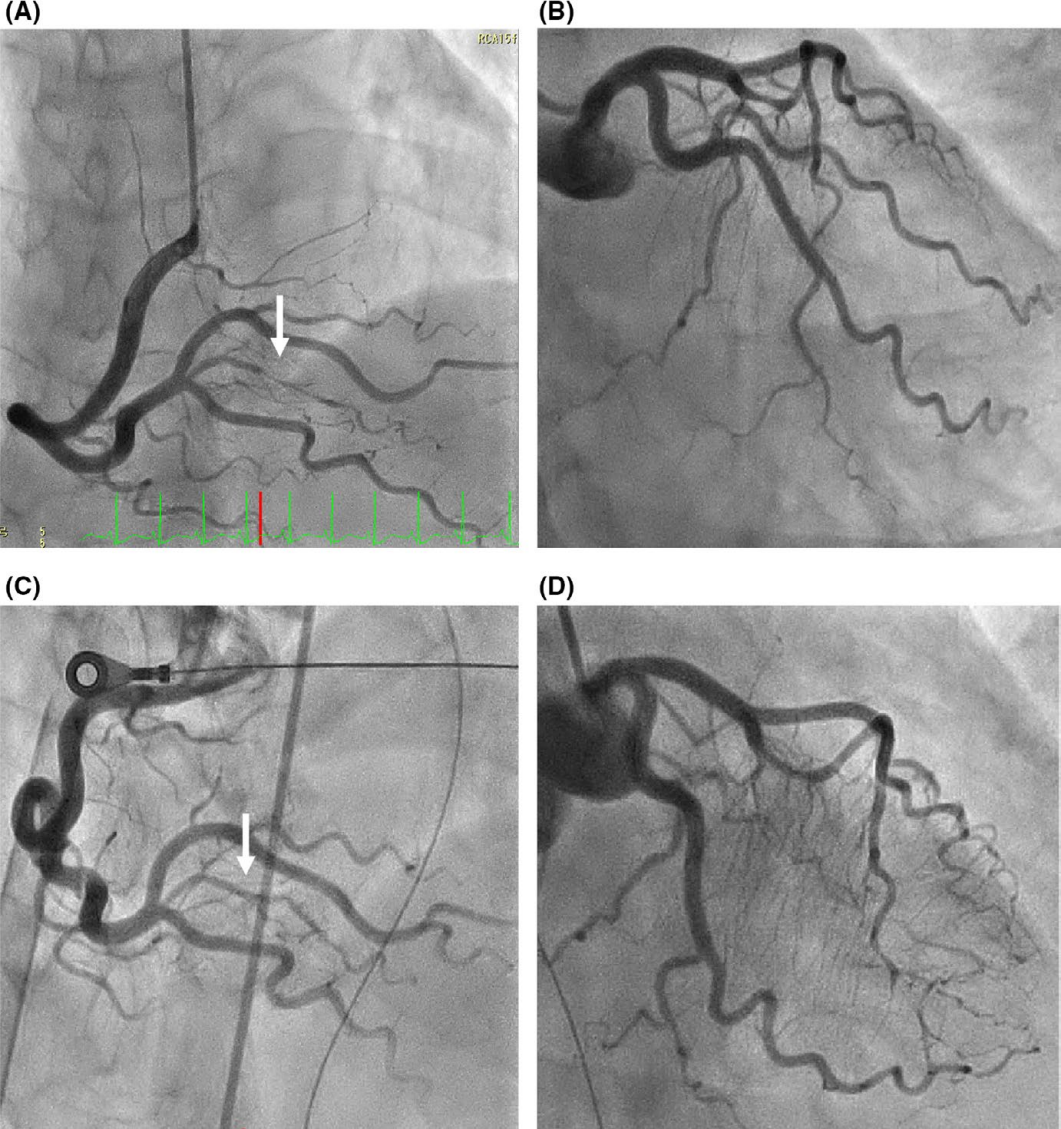
Angiogram of the SCAD in right coronary artery distal (upper white arrow) in 2015 (A) and the healed finding (lower white arrow) in 2018 (C). Angiogram of left coronary artery in 2015 (B) and 2018 (D)

A 57‐year‐old woman, who had a history of SCAD in the distal right coronary artery (RCA) and continued undergoing conservative therapy, was admitted to our hospital. Three years ago, she visited our hospital for typical chest pain. The electrocardiogram revealed ST‐segment elevation in the inferior leads (Figure 1A). The coronary angiography (CAG) showed 90% luminal stenosis in the distal RCA (Figure 2A) and no significant left coronary artery stenosis (Figure 2B). She had no history of diabetes mellitus, dyslipidemia, autoimmune disease, vasculitis, and smoking habit. She also had no family history of connective tissue diseases, such as Marfan's syndrome and Ehlers‐Danlos syndrome. After 7 days of observational admission, she continued to visit the outpatient department and was prescribed a calcium channel blocker.

Surprisingly, she presented with cardiopulmonary arrest (CPA) and return of spontaneous circulation (ROSC) in the ambulance and was brought to our emergency department. After ROSC, her systolic blood pressure was 120 mm Hg and her pulse was 80 bpm (sinus rhythm); however, her level of consciousness decreased (E1V1M1). She displayed agonal gasp, and we performed intubation with an artificial ventilator. The electrocardiogram showed tachycardia and ST‐segment elevation in the lead I and aVL (Figure 1B). A chest X‐ray showed moderate cardiomegaly and pulmonary congestion. The laboratory data showed that AST 521 IU/L, ALT 695 IU/L, LDH 1546 IU/L, Cr 1.01 mg/dL, creatinine kinase‐muscle/brain was elevated to 56 IU/L, and high‐sensitivity troponin I was 1551 pg/mL. The echocardiogram revealed pericardial fluid and reduced systolic function, and the left ventricular ejection fraction was 40%. In the angiography room, she showed low blood pressure and bradycardia, so we administered noradrenaline, adrenaline, and atropine. However, her hemodynamics collapsed, so we introduced percutaneous cardiopulmonary support (PCPS) and intra‐aortic balloon pumping (IABP). Emergent CAG revealed TIMI3 flow (Figure 2C,D). The distal RCA, which had past history of focal SCAD 3 years ago, showed good flow to the distal coronary artery (Figure 2C, arrow). From CAG results, we administered a conservative treatment. Despite our efforts, she suffered a cardiac arrest and unfortunately died on the same day.

**FIGURE 3:**
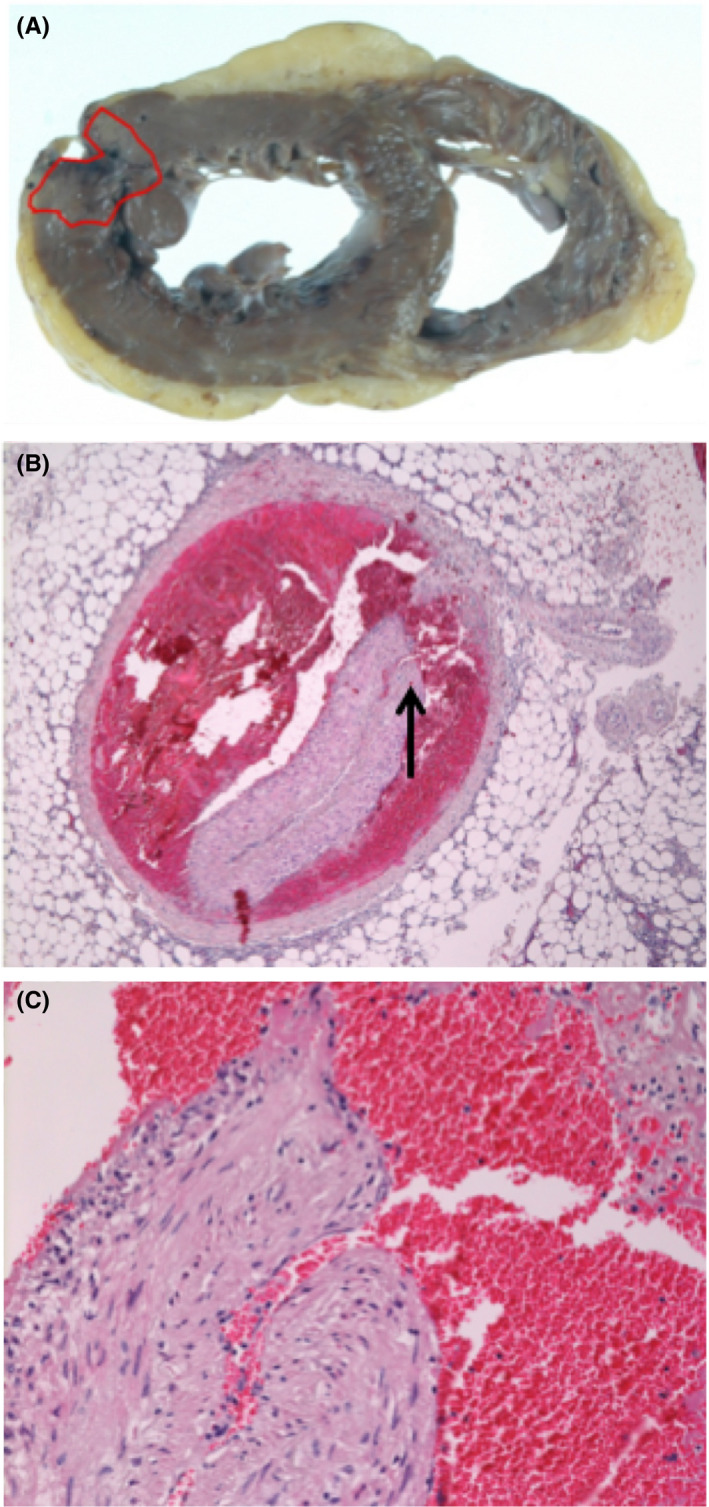
Histopathological appearance (hematoxylin and eosin statin) of the myocardium and left anterior descending branch. A, Acute ischemic lesion (surrounded by a red line) in myocardium. B, The arrow indicates an intimal tear which communicate between the true lumen and false lumen. Intraluminal hemorrhage compressed the true lumen in left anterior descending branch. C, An intimal tear in high power field

Her postmortem examination revealed extensive acute myocardial infarction (AMI) involving a part of the left ventricular free wall rupture (Figure 3A). IMH in the false lumen, over proximal to the middle of the left anterior descending artery (LAD), was also noted compressing the true lumen (Figure 3B). Moreover, we discovered an intimal tear that communicated with the true lumen and the false lumen in the proximal LAD (Figure 3C), it was not detected by CAG. These findings indicated that the onset of the intimal tear had caused blood flow to the false lumen and IMH formation compressing the true lumen and, thus, leading to AMI.

## 3 DISCUSSION

According to previous research, SCAD is characterized by the spontaneous formation of IMH within the wall of a coronary artery. The separation occurs in the outer third of the tunica media and IMH filling in the false lumen compresses the true lumen, leading to coronary insufficiency and myocardial ischemia.^3^ There are two possibilities for the formation of IMH with SCAD. The first theory proposes that the primary pathological event is a disruption of the tunica intima of a coronary artery that allows blood from the true lumen flow into the false lumen. The other theory proposes that the primary pathological event is a braking of a vasa vasorum, which is within the vessel wall and provides the vessel with blood.^3^, ^5^ In this case, the tear found in the coronary artery may be an entry point of the dissection and is called an “intimal tear.”

The SCAD recurrence within 30 days after first onset was reported in 10%‐20%^6^ and long‐term recurrent rates in 27% at 4‐5 years.^7^ The use of beta‐blockers was associated with lower risk of SCAD.^8^ It has been reported that the early hormone replacement therapy (HRT) for postmenopausal women reduced cardiovascular events.^9^ HRT might be useful management for secondly prevention after SCAD for postmenopausal women. However, the clinical predictors of recurrence of SCAD are not established, further studies should be explored.

In conclusion, we report the angiographic findings of a healed SCAD of the distal RCA occurring 3 years ago and show the pathological findings of an intimal tear that lead to SCAD of the proximal LAD. Consequently, SCAD patients are should be closely followed and further randomized trials are necessary to formulate clinical guidelines for preventing the recurrence of SCAD.

## CONFLICT OF INTEREST

None declared.

## AUTHOR CONTRIBUTIONS

JK, ST: involved in preparing and writing the manuscript. ST: involved in the angiology and revision of the manuscript. All authors approved the final version of the case report for submission to *Clinical Case Reports*.

## References

[1] Saw J , Mancini GBJ , Humphries KH . Contemporary review on spontaneous coronary artery dissection. J Am Coll Cardiol. 2016;68(3):297‐312. 27417009 10.1016/j.jacc.2016.05.034

[2] Saw J , Humphries K , Aymong E , et al. Spontaneous coronary artery dissection clinical outcomes and risk of recurrence. J Am Coll Cardiol. 2017;70(9):1148‐1158. 28838364 10.1016/j.jacc.2017.06.053

[3] Hayes SN , Kim ESH , Saw J , et al. Spontaneous coronary dissection: current state of the science. Circulation. 2018;137:e523‐e557. 29472380 10.1161/CIR.0000000000000564 PMC5957087

[4] Williams JK , Armstrong ML , Heistad DD . Vasa vasorum in atherosclerotic coronary arteries: responses to vasoactive stimuli and regression of atherosclerosis. Circ Res. 1988;62(3):515‐523. 3342475 10.1161/01.res.62.3.515

[5] Yip A , Saw J . Spontaneous coronary artery dissection ‐a review. Cardiovasc Diagn Ther. 2015;5(1):37‐48. 25774346 10.3978/j.issn.2223-3652.2015.01.08 PMC4329168

[6] Saw J , Humphries K , Aymong E , et al. Spontaneous coronary artery dissection: clinical outcomes and risk of recurrence. J Am Coll Cardiol. 2017;70:1148‐1158. 28838364 10.1016/j.jacc.2017.06.053

[7] Shufelt CL , Pacheco C , Tweet MS , Miller VM . Sex‐specific physiology and cardiovascular disease. Adv Exp Med Biol. 2018;1065:433‐454. 30051400 10.1007/978-3-319-77932-4_27 PMC6768431

[8] Main A , Prakash R , Starovoytov A , et al. Characteristics of extension and de novo recurrent spontaneous coronary artery dissection. EuroIntervention. 2017;13:e1454‐e1459. 28891472 10.4244/EIJ-D-17-00264

[9] Nakashima T , Noguchi T , Haruta S , et al. Prognostic impact of coronary artery dissection in young female patients with acute myocardial infarction: a report from the Angina Pectoris‐Myocardial Infarction Multicenter Investigators in Japan. Int J Cardiol. 2016;207:341‐348. 26820364 10.1016/j.ijcard.2016.01.188

